# Chicago Society of Ophthalmology and Otology; Meeting December 14th

**Published:** 1887-02

**Authors:** 


### SOCIETY REPORTS.

---

THE CHICAGO SOCIETY OF OPHTHALMOLOGY AND OTOLOGY *met December 14th*, at the Tremont House. The President, E. L. HOLMES, M.D., in the chair.

Drs. Gardiner, Holmes and Colburn were appointed a committee on membership.

The following paper was read by LYMAN WARE, M.D.:

### ON LUPUS VULGARIS.

*History.*—This is no new disease; but on account of its frequent occurrence, and, until the past few years, its almost incurability, I beg to call the attention of the society to a few cases which have come under my observation. Before referring to the history of these cases, or submitting to your examination two or three patients who have consented to appear before you, I would, in a few words, recall to your minds some notable features of this disease. There is no doubt that it was generally recognized at least 5,000 years ago, and designated "Lupus." That it may have been confounded by ancient writers, in many cases, with carcinoma is highly probable. Willan was the first to apply the term lupus exclusively to certain forms of ulceration about the face, while Bateman made no actual distinction between the effects and course of carcinoma and lupus, except that he considered the latter curable by arsenic, the former not.

*Symptomatology.*—Lupus varies much in appearance, according to the age of the patient, constitution, state of health, kind of diet, and the part affected. At first it appears in the form of tubercles, either isolated or in groups, which may be deeply imbedded in the true skin, beneath it, or quite superficial, and always spreads by infecting the adjacent skin. It is never congenital, though it may appear early in life. Unchecked by treatment it increases more or less steadily for many years. At times the disease may appear checked for years, when it will again break out with renewed vigor. In old age the severity of the disease is often diminished. Lupoid tuburcles may terminate in involution, leaving the skin atrophied and glistening like a scar, or in disintegration and ulceration. It occurs upon any portion of the body, most frequently, by far, upon the face. The cases which have come under my observation originated on the lower eyelid, extending gradually to the eye and nose. Lupus of the ala nasi and the tip of the nose assumes the disseminate form. The tubercles are prominent, and coalescing form large, irregular protuberances. Lupus on the lower lid produces first complete ectropion, in consequence of cicatricial contraction of the skin, then gradually extends to the palpebral conjunctiva, the ocular and deeper tissues of the eye, involving, at last, the entire orbit. As the disease involves the conjunctiva it imparts to it a dark, reddish-brown color, and is decidedly trachomatous in appearance. Later, it becomes smooth, glistening and atrophied.

*Etiology.*—Many theories have been advanced respecting the cause of lupus. Both ancient and modern writers refer it to scrofula or syphilis. Although the terms scrofula and scrofulous have been used for ages, by physicians as well as laymen, throughout the entire world, it must be confessed we have no very clear idea as to what scrofula is, or to what exact condition scrofulous should be applied. In my younger days I was taught that scrofula, being a literal translation of the Latin scrofa, swine, was a disease common, to those who eat pork. I have since thought that such an explanation was unfounded, and that the term "scrofula" originated in the fact that the sub-maxillary furrow, which does not exist in well-fed swine, was obliterated by glandular enlargement. Some who hesitate to use the word scrofula, designate exactly the same condition by the term "struma," or "strumous diathesis."

Although we may not be able to define clearly the term "scrofula," or "struma," yet in a case of indolent swelling of the lymphatic glands, large lips, flabby, soft muscles, with a feeble constitution, and where the slightest irritation produced chronic inflammation, followed by suppuration, we would not hesitate to designate such a condition a scrofulous or strumous. In most of the cases that came under my observation such a condition was not present, nor was there any history of syphilis. Some writers, though not considering lupus a syphilitic disease, think it shows a syphilitic taint; yet I was not able to discover a single symptom that pointed toward hereditary syphilis. The patients generally were in excellent health, appetite and digestion good. The more I compare the two diseases the less resemblance I find. Lupus is an exceedingly chronic, indolent disease, arising from small tubercles imbedded in the substance of the skin, does not become rapidly worse, may persist for years without producing constitutional disturbance, and is wholly unaffected by anti-syphilitic treatment. It is also essentially a disease of early life, seldom making its appearance before the third year, and hardly ever after the twentieth or twenty-fifth. When it appears later it will generally be found to be a recurrence, and not a primary attack. The treatment of lupus rather indicates that it may be allied to the malignant diseases, a connecting link, as it were, between the non-malignant and malignant.

Volkmann has recently laid great stress upon the fact that every portion of the diseased tissue should be most thoroughly eradicated by the scoop; and the same thing has been taught and attempted for many years by means of the knife, the caustic, or the galvano-cautery. It has long been insisted that whatever method be adopted, it must be thoroughly and unflinchingly carried out, and repeated from time to time, if necessary, according to the exigencies of the case, or its tendency to relapse. Most authors have considered it necessary to treat the disease locally—some by constitutional remedies only. In the cases reported I have tried eaeh method separately, then unitedly, and I have no hesitancy in saying that when the local treatment was combined with proper internal medication, the advance toward recovery was much more rapid and more satisfactory than when one method only was followed. The internal treatment consisted principally of tonics, as quinia, iron, strychnia, phosphates, and particularly arsenic, either in the form of arsenious acid or Fowler's solution, and special attention to a plain, nutritious diet, and out-door life. The local treatment consisted in the complete destruction of the existing lupoid tubercles, in whatever state of development, by means first of the scoop or Valkmann's curette, then by a thorough and prolonged application of pyrogallic acid in crystals. In some cases, after removing the superficial layer; consisting of morbid products, scales, secretions, etc., a pledget of absorbent cotton was saturated in a 4 per centum solution of cocaine, and firmly held on the diseased tissue. In other cases, where the lupoid tubercles were deeply imbedded, or where the disease was at all extensive, general anæsthesia was first induced. The patients never complained so much of the acid application as of the cutting or scooping out of the lupoid mass. The pyrogallic acid uniting with the blood and lupoid tubercles produces a thick, brownish, syrupy substance, and as long as this is produced the acid should be applied. Jarisch, of Vienna, was the first to call the attention of the medical profession to the local use of pyrogallic acid, though it had long been used in the arts, particularly in photography. Dr. A. Vesey (Dublin Jour. Med. Sci. 1878), nine or ten years since, observing its remarkable astringent effect on the hands of those engaged in photography, was led to employ it in internal hemorrhage, and especially in the hæmoptysis of phthisis, in which he found it very efficient, in grain doses, frequently repeated (every hour or oftener). Jarisch first used it in the form of a ten-per-centum ointment, with vasaline. Besnier, of Paris, makes use of a saturated solution of the acid in ether; applies it with a camel's-hair pencil to the diseased portion, and at once covers this with a layer of traumaticine. These applications are repeated until the disease is thoroughly eradicated.

Schwimmer, of Budapest, after destroying the lupoid tubercles, by means of the ten-per-centum ointment already mentioned, prevents a return of tubercles in the cicatrix, which is of frequent occurrence, by the application of mercurial plaster. In some few cases where pyrogallic acid has been used extensively, and long continued, poisoning has resulted; but where its use is restricted to the removal, either of lupus or epithelioma, it is not at all likely to be complicated by any such unpleasant results. In cases of poisoning the kidneys are first affected, prostration and febrile disturbances appear, the urine becomes brownish or olive green in consequence of hæmoglobinuria, the skin tinged with green, and a glairy mucus is vomited. Dr. Charles W. Allen, of New York, has reported a number of cases of lupus treated by pyrogallic acid, and has expressed himself as much pleased with its effect. He made use of both the powder and ointments of varying strength, made with vasealine. He considers the acid of great value, because it specially attacks the lupoid tubercles, induces destructive ulceration in and about them, leaving the healthy tissue unchanged. In any preparation of pyrogallic acid care must be exercised not to combine it with an alkali, which would neutralize it, nor with a metal, which it would reduce. Six cases have been treated, but in case No. 1 iodoform, in powder, was used instead of pyrogallic acid.

Jas. M., æt. 45 years, was first attacked twenty years since, while in the U. S. army. The disease made its appearance at the inner canthus of the right eye. He attributed it to an injury received while riding under a tree, and being struck by an overhanging limb. The disease gradually extended to the under eyelid, involved a portion of the upper, encroached upon the cheek, and finally the deeper tissues of the eye itself. The patient was given ether, and the diseased mass thoroughly removed by means of the curette, and the wound dressed with powdered iodoform. Two weeks subsequently a few lupoid tubercles again made their appearance at the inner canthus, but were easily removed by the curette without anæsthesia. The patient made a rapid recovery, and remained under observation about six months, and no relapse occurred. He assured me, on leaving, that should there be a return of the disease he would let me know. He was placed, from the first, upon Fowler's solution of arsenic, and advised to continue its use, for at least six months. The photographs which I show give a good idea of the appearance of the case before and after treatment.

CASE II. F. W., æt. 50, residence 44 Desplaines street, Chicago, stonecutter. Twenty years since a small tubercle, about the size of a pea, made its appearance upon the left ala nasi. It slowly increased, until it attained the size of a large bean, ulcerating and extending at its edges. A few years later lupoid tubercles appeared at the inner canthus of the left eye, and gradually extended to the lower lid. Mr. W. had submitted to a great variety of treatment, which had only partially kept the disease in check. When he came under observation the protuberance on the ala nasi was, at least, the size of a large lima bean, dark red, and ulcerated at the edges. The ulceration at the inner canthus occupied an irregular space, about the size of a 10-cent piece. Although there was at least an inch of perfectly healthy skin and tissue intervening between the two, the patient was confident there was some connection between them. When the ulcer of the nose was red and painful, that of the eye was also worse. As the patient had considerable fortitude the lupoid tubercles of both nose and eye were thoroughly curetted, without the aid of ether, and the pyrogallic acid applied until the brownish, syrupy substance ceased to form. The history of this case differed somewhat from the others, in this respect: that for years there had been more or less pain. In fact, of late the pain had been so great that it was impossible for him to work at his trade, or sleep, sometimes for several nights in succession. The operation relieved him completely of the pain, and he was able to sleep, and returned at once to his work. Several operations have been necessary, but he has been able to sleep regularly and work constantly, and at the present time appears to be completely cured.

CASE III.—Minnie, æt. 16. The lupoid ulceration first began when she was twelve years of age, unaccompanied by any pain. When she first came under treatment the entire upper and lower lids, and about one-third of the adjacent cheek, were involved. Ether was administered, the entire ulcerated surface was curetted, and the acid applied. This case, being at the Illinois Charitable Eye and Ear Infirmary, and my term of service about expiring, came under the charge of my colleague, Dr. Gardiner, who continued the same course of treatment. After remaining a month or two in the institution, and no indication of the disease reappearing, she was allowed to return to her home. Six months have now elapsed, and there has been no return of the disease; but as there is slight ectropium of the lower lid, in consequence of the deep ulceration, a plastic operation will be necessary to restore it to its normal condition. In all cases of lupus about the eye I have considered it prudent to protect the cornea from the action of the acid by means of a thick layer of absorbent cotton. I have now used the pyrogallic acid in five cases, and in three or four very extensively and freely, yet in no case have I seen any symptoms which would indicate poisoning.

THE SUBJOINED REPORT OF A CASE OF "RECURRENT HEMORRHAGE INTO THE ANTERIOR CHAMBER," WAS READ BY BOERNE BETTMAN, M.D.

Cases of concussion of the eyeball, with subsequent hemorrhage into the anterior chamber, are not uncommon. Such occurrences are frequently noted down in our journals, and are alluded to in our text-books as uninteresting matter of fact. The case I wish to bring to the society's notice to night differs so much from the ordinary ones that its presentation need not be prefaced with an excuse.

On the 19th of May, 1886, Mr. and Mrs. W. hurriedly entered my office with their little daughter Ettie, aged four. They informed me that the child, whilst playing "Indians" with her comrades, was struck in the left eye, by a projectile propelled from a toy gun in the hands of a boy. The projectile was a twig of a tree. The end was apparently smooth, to judge from the appearance of the contused lid. The stick could not be found. The propelling force was a broad band of rubber, kept tense by a wooden trigger. The gun was within a few feet of the child when the accident occurred, three-quarters of an hour before I saw the patient. On examination I found the following condition: The upper right lid was œdematous and reddened. On lifting the latter, the eye was found bathed in tears, and the conjunctiva slightly injected; cornea normal. The whole anterior chamber was filled with blood. In the lower part it formed a large clot, hiding the iris from view. The pupil was dilated and elongated in the vertical diameter. An ophthalmoscopic examination could not be made. The child was unable to count fingers close to the eye. I ordered cold compresses and instillation of two drops, every few hours, of a one-per-centum solution of atropine. When I again saw the child, about three hours later, the pupil was dilated ad maximum, and the greater part of the hemorrhage was absorbed. Fingers were readily counted. On the morning of the 20th but a small clot of blood remained, in the lower part of the anterior chamber. At the next visit, May 21, I learned from the mother that the patient had passed a very restless night, occasioned by spells of sudden, intense pain. The first attack occurred in the evening. During a momentary absence of the parent the child left its bed and ran to the window, which it opened. Hearing its mother return it hastily essayed to regain its bed, but stumbled and fell heavily to the floor. The fall was followed by severe pain in the injured eye. A second attack took place at midnight, and a third early in the morning. I found the eye in a glaucomatous state. Tension 11, very marked ciliary injection, anterior chamber full of blood. The eye was rebandaged, and cold applications applied over the roller. A one-per-centum solution of eserine was prescribed, two drops to be instilled into the eye five times a day. The child was kept quiet in bed. In the evening I found a great improvement. Tension reduced, but still higher than normal. Hemorrhage mostly absorbed. At midnight the child was again awakened by the excruciating pain in the left eye. The morning showed a return of yesterday's condition:

extensive hemorrhage and increased tension. These sudden attacks of severe pain came on without any apparent restlessness on the part of the child. Whilst in the midst of a sound sleep it would be suddenly awakened by the darting pains. hesitated to reinstill the eserine, thinking that the action of the drug favored a separation of the partly healed iris, which I supposed had been lacerated at the lower border, covered by the blood-clot. The child had been kept quiet in bed. Both eyes were bandaged, for a time; the right one constantly. Coffee and other stimulants were forbidden. In fact, everything had been done to favor a rapid union of the parts. I decided to try the effects of ergot, and ordered the fluid extract to be given, in twelve-drop doses every three hours, in order to reduce the action of the heart, and permit a sufficiently firm organization of the blood-clots in the lacerated blood-vessels. The introduction of this remedy was soon followed by good effects. The blood disappeared entirely. The pupil remained for a time elongated, but later on resumed its normal shape.

